# Application of Green Gold Nanoparticles in Cancer Therapy and Diagnosis

**DOI:** 10.3390/nano12071102

**Published:** 2022-03-27

**Authors:** Saman Sargazi, Ushna Laraib, Simge Er, Abbas Rahdar, Mohadeseh Hassanisaadi, Muhammad Nadeem Zafar, Ana M. Díez-Pascual, Muhammad Bilal

**Affiliations:** 1 Cellular and Molecular Research Center, Research Institute of Cellular and Molecular Sciences in Infectious Diseases, Zahedan University of Medical Sciences, Zahedan 98167-43463, Iran; sgz.biomed@gmail.com; 2 Department of Pharmacy, College of Pharmacy, University of Sargodha, Sargodha 40100, Pakistan; ushnalaraib@yahoo.com; 3 Department of Biochemistry, Faculty of Science, Ege University, Izmir 35100, Turkey; simgeer89@gmail.com; 4 Department of Physics, University of Zabol, Zabol P.O. Box 98613-35856, Iran; 5 Department of Plant Protection, Shahid Bahonar University of Kerman, Kerman 76184-11764, Iran; mhassanisaadi@yahoo.com; 6 Department of Chemistry, University of Gujrat, Gujrat 50700, Pakistan; znadeempk@gmail.com; 7 Universidad de Alcalá, Facultad de Ciencias, Departamento de Química Analítica, Química Física e Ingeniería Química, Ctra. Madrid-Barcelona, Km. 33.6, Alcalá de Henares, 28805 Madrid, Spain; 8 Huaiyin Institute of Technology, School of Life Science and Food Engineering, Huaian 223003, China; bilaluaf@hotmail.com

**Keywords:** biosynthesis, gold nanoparticles, green synthesis, surface plasmon resonance, leaf extract, toxicity

## Abstract

Nanoparticles are currently used for cancer theranostics in the clinical field. Among nanoparticles, gold nanoparticles (AuNPs) attract much attention due to their usability and high performance in imaging techniques. The wide availability of biological precursors used in plant-based synthesized AuNPs allows for the development of large-scale production in a greener manner. Conventional cancer therapies, such as surgery and chemotherapy, have significant limitations and frequently fail to produce satisfying results. AuNPs have a prolonged circulation time, allow easy modification with ligands detected via cancer cell surface receptors, and increase uptake through receptor-mediated endocytosis. To exploit these unique features, studies have been carried out on the use of AuNPs as contrast agents for X-ray-based imaging techniques (i.e., computed tomography). As nanocarriers, AuNPs synthesized by nontoxic and biocompatible plants to deliver therapeutic biomolecules could be a significant stride forward in the effective treatment of various cancers. Fluorescent-plant-based markers, including AuNPs, fabricated using *Medicago sativa*, *Olax Scandens*, *H. ambavilla*, and *H. lanceolatum*, have been used in detecting cancers. Moreover, green synthesized AuNPs using various extracts have been applied for the treatment of different types of solid tumors. However, the cytotoxicity of AuNPs primarily depends on their size, surface reactivity, and surface area. In this review, the benefits of plant-based materials in cancer therapy are firstly explained. Then, considering the valuable position of AuNPs in medicine, the application of AuNPs in cancer therapy and detection is highlighted with an emphasis on limitations faced by the application of such NPs in drug delivery platforms.

## 1. Introduction

Cancer is the uncontrolled proliferation of a healthy cell that creates genetic abnormalities and alterations which accumulate within cells and tissues, leading to tumorigenesis (the development of cancerous cells) [1]. Indeed, this disease is a malignant tumor with a high death rate, and millions of people worldwide die each year from various varieties of cancer [2]. According to World Health Organization (WHO), lung cancer now ranks sixth among the leading causes of death globally [3]. The occurrence of cancer is also associated with environmental factors, including air pollution, exposure to chemical pesticides, heavy metals, radiation, and infectious agents [4].

Several techniques, including computed tomography (CT), color Doppler ultrasound imaging (CDI), positron emission computed tomography (PET), magnetic resonance imaging (MRI), and tumor biomarkers, are popular methods that have been widely used to detect cancer [5,6,7,8]. However, the low sensitivity and poor accuracy of these techniques restrict their use in early cancer detection [2]. Overall, diagnosed cancers are treated with seven conventional methods, namely surgery, immunotherapy, radiotherapy, chemotherapy, targeted therapy, hormone therapy, and bone marrow transplantation [9]. Surgical techniques and chemotherapy have been the initial treatment choices for cancer patients [10]. However, the size of tumors and the spread of metastases to nearby tissues restrict the selection of the treatment method for carcinoma cells [11]. Targeted therapy can be accomplished through various pathways, including selective photothermal absorption or targeted drug delivery [12,13], and causes fewer side effects and better treatment effectiveness due to specific treatment of cancer cells [9]. Therefore, novel medications for targeted therapy have garnered increasing interest in recent decades.

**Figure 1:**
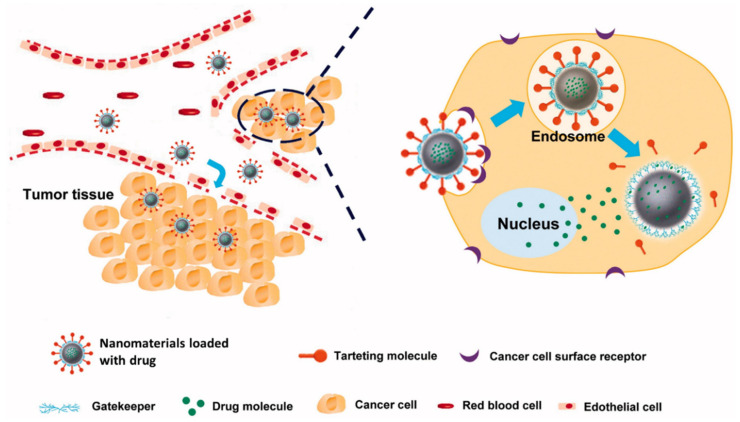
Schematic illustration of targeted drug delivery system. Reprinted with permission from Ref. [18]. Copyright 2017 Taylor & Francis.

Nanotechnology is a rapidly growing area that utilizes nanoscale materials for diagnosis and treatment goals. Cancer therapy is one medical area in which nanomaterials (NMs) have a variety of applications [14]. In this context, the integration of nanoscience and pharmaceutical science can pave the way for a significant change in medical science, including establishing drug delivery systems for the targeted therapy of cancer. Numerous nanostructures, such as gold nanoparticles (AuNPs), carbon nanotubes (CNTs), dendrimers, liposomes, and micelles, are often used as delivery vehicles for targeted and controlled drugs [15,16]. Size, shape, hydrophobicity, surface properties, and other physicochemical characteristics of these drug delivery vehicles have affected their efficiency in drug delivery [17]. The diverse variety of surface chemistries of NMs creates great potential for them to couple with molecular targeting agents such as antibodies, allowing them to target tumor tissue and cause it to die specifically. In targeted drug delivery, cell surface receptors, such as monoclonal antibodies or therapeutic antibodies, can recognize carcinoma cells. Released medicines regulate the downstream cell cycle progression and induce cell death following their attachment to cancer cells. Figure 1 depicts the schematic illustration of the targeted drug delivery system based on NPs [18].

Gold nanoparticles (AuNPs) have been applied in targeted therapy for targeted drug delivery [19]. Undoubtedly, using a controlled drug delivery system provides a fundamental approach for improving the therapeutic effects of drugs and decreasing the side effects of drug molecules [9,20]. AuNPs have also been utilized as delivery vehicles in combined photothermal therapy [21] and for the effective delivery of drugs to cancer cells [22].

The application of materials derived from natural products to treat various human ailments, including cancers, is rapidly growing [23,24]. Strong evidence has suggested that natural resources can display cancer chemopreventive and therapeutic properties [25,26,27,28]. Despite tremendous advancement in cancer research, secondary compounds derived from natural resources have been identified as worthwhile cancer therapeutic candidates [29]. Indeed, in utilization time, phytochemicals are confronted with various chemical and physical obstacles. Such circumstances can change their natural structure and impair their anticancer efficacy [30]. These obstacles increase the need to use alternative strategies to overcome these limitations. Therefore, developing novel formulations and nano-based drug delivery systems is considered promising.

Possible hazards, such as carcinogenicity and undesirable toxic effects, restrict the use of NPs in various applications, especially in biomedicine [31,32]. The biological synthesis of NPs is achieved using reducing agents derived from various bioresources, including plants [33,34], yeasts [35], bacteria [36], actinomycetes [37], fungi [38], and algae [39]. The biological synthesis of magnetic nanoparticles (MNPs) provides safe and effective medication carriers and diagnostic agents for cancer therapy goals [40,41]. In this regard, biogenic AuNPs are reported to have significant medical applications as drug carriers due to their simplicity of synthesis, negligible toxicity, and stability [41]. Conventional medicines used for cancer treatment are toxic to the body, causing side effects or untargeted effects on healthy cells, developing drug-resistant cells, rapid drug metabolism, and shortening effective treatment duration [41]. Green synthesis mediated by plants provides the potential to synthesize NPs solutions containing biologically active molecules derived from natural extracts, which can have an effective anticancer activity on human cancer cells. It has been demonstrated that a significant number of biosynthesized AuNPs display anticancer activity; however, their efficacy and cellular effects rely on the biological extract used during the synthesis technique [42]. AuNPs prepared using *Corchorus olitorius* extract as a stabilizing and reducing agent displayed significant anticancer effects against three cancer cell lines, including colon cancer HCT-116, breast adenocarcinoma MCF-7, and hepatocellular cancer HepG-2 [43]. Green synthesized AuNPs can also be used for diagnostic goals in targeted therapy. In the study by Fazel et al. [44], the anticancer effect of AuNPs biosynthesized by *cocoa* extract was evaluated. Biogenic AuNPs possess the potential ability to absorb near-infrared (NIR) and can be promising candidates for diagnostic routes in photothermal therapy of cancers.

Considering the valuable position of AuNPs in medicine, this review focuses on the application of AuNPs in cancer theranostics with an emphasis on limitations faced by the application of such NPs in drug delivery systems.

## 2. AuNPs; In Vitro Characterization (In Vitro)

Characterization of biogenic AuNPs is a crucial step prior to their use in medical applications. The characterization of AuNPs’ shape, surface topology, size distribution, monodispersity, and stability will provide valuable knowledge on the properties of AuNPs as well as insight into their controllable production for therapeutic applications. After the color change of the solution as a resulting reduction of metal ions to metal nanoparticles, the formation of NPs is monitored by several analyses. Below, standard techniques for the characterization of NPs, such as AuNPs, are outlined.

UV-Visible spectroscopy is the initial instrumental method to confirm the synthesis of AuNPs [45]. The surface resonance generated by electron oscillations is in the 500–600 nm wavelength range [46]. The size, shape, and concentration of the AuNPs impact the wavelength at which the absorbance peak emerges and the absorbance peak intensity [47]. Larger particles shift the absorbance peak to longer wavelengths (600 nm) and increase the peak intensity [41]. The morphology, topography, and geometry of AuNPs are considered fundamental properties and strongly condition their therapeutic characteristics. Scanning electron microscopy (SEM), field-emission scanning electron microscopy (FESEM), and transmission electron microscopy (TEM) can provide information on the morphology and surface features of the AuNPs. SEM and FESEM scan the NP surface with a beam of electrons and provide information about the surface topography and composition of the sample. In contrast, TEM uses transmitted electrons and offers insight into size and shape [48,49,50]. In addition, TEM, SEM, and FESEM analyses have been used to estimate the NP size [51,52,53]. However, the most common technique to estimate AuNP size is dynamic light scattering (DLS), which offers information about the hydrodynamic diameter of AuNPs in the aqueous phase. Diameter values obtained by SEM/TEM are typically smaller than those derived from DLS measurements. According to Erjaee et al. [54], these discrepancies might arise from the fact that microscopic techniques only evaluate the particles’ physical size in the solid-state, but DLS analyzes the particles’ hydrodynamic diameter, which is the effective diameter in a liquid medium, and ligands attached to the NP can affect its Brownian motion, which influences the diffusion of the particles and hence directly effects the size estimation of DLS [55]. The crystalline structure of AuNPs is characterized by X-ray diffraction (XRD), and the surface composition is characterized using energy-dispersive X-ray (EDAX or EDS) analysis [56,57]. Fourier transformation infrared spectroscopy (FTIR) is a versatile, cost-effective, and simple technique to determine the biomolecules involved in the bioreduction of metallic ions [58]. The stability of AuNPs in a solution is conditioned by the biomolecules that form a protective coating surrounding each particle. The thickness of this layer determines the NP’s stability [41].

## 3. Prospective Application of Plant-Based Materials in Cancer Therapy

**Figure 2:**
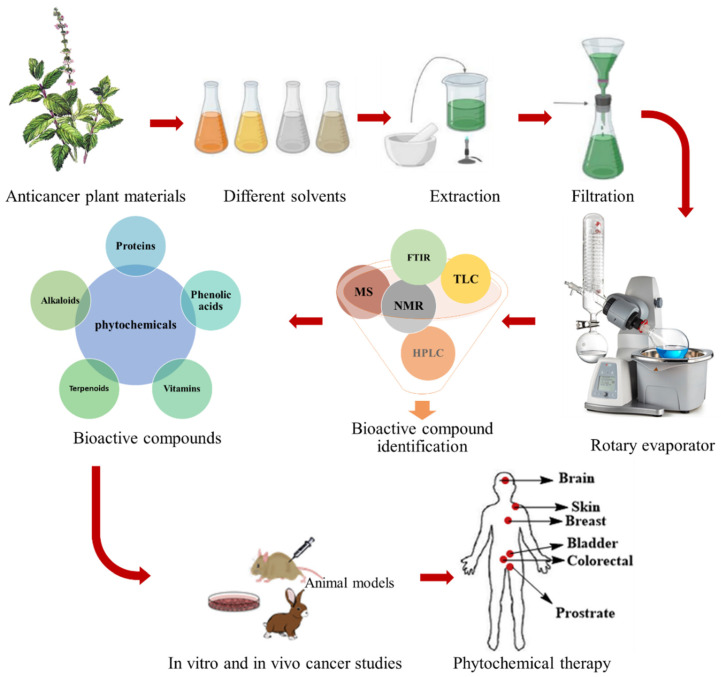
Schematic process of identification, characterization, and future application of herbal plants in cancer therapy.

Among natural resources, plants, due to their huge variety with influential phytochemicals and therapeutic properties, have a crucial role in treating many ailments worldwide, including cancer [59]. According to scientific records, over 80% of the world’s population relies on plant-derived pharmaceuticals for health, and in addition, medicinal plants have played a critical role in extending life span [60,61]. In some plants, the therapeutic properties can relate to low-molecular-mass compounds known as secondary metabolites. Terpenoids, alkaloids, and phenolics are the main groups of secondary metabolites [62,63]. Certain plant species can play a crucial role in blocking or activating transduction pathways in live cells by creating associated secondary metabolites with antimutagenic and cancer capabilities [64]. Some plant-derived anticancer drugs include vincristine [65], emodin [66], paclitaxel [67], kaempferol [68], linalool [69], colchicine [70], rutin [71], and quercetin [72]. Although the number of plant-derived anticancer compounds is vast, only a few have reached clinical use after successfully navigating the lengthy, expensive, and bureaucratic path from identification to cancer therapy effectiveness [64]. In their publication, Iqbal et al. [1] extensively documented some important medicinal plants and their anticarcinogenic phytochemicals against a particular type of cancer. Additionally, Ijaz et al. [73] specifically listed the plant-derived anticancer compounds against various skin carcinoma cell lines. Figure 2 illustrates a schematic process for anticarcinogenic phytochemical synthesis, characterization, and potential applications as cancer treatment agents.

Despite considerable therapeutic advantages, the efficacy of natural products is compromised by their poor stability, low aqueous solubility, limited bioavailability, and short retention period; all of these factors restrict their therapeutic applicability [30,74].

## 4. Green Synthesis of AuNPs

**Figure 3:**
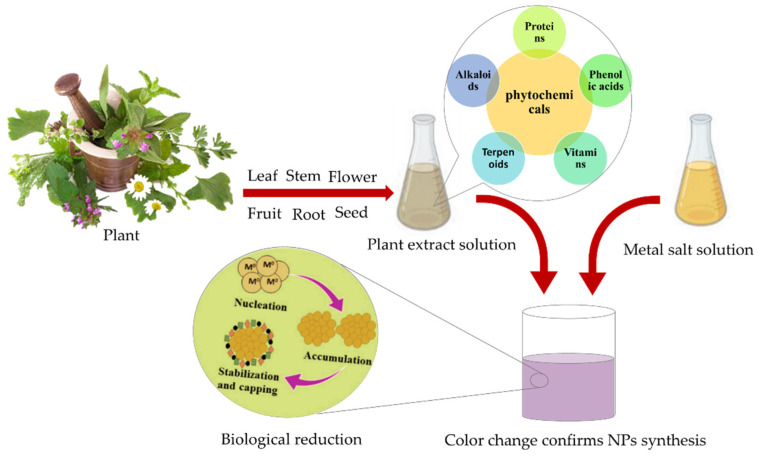
Schematic illustration of biosynthesis of metallic NPs from a plant.

Phytochemical compounds in plants, such as proteins, organic acids (including fatty acids and phenolic acids), vitamins, carbohydrates, alkaloids, and secondary metabolites, serve as reducing, stabilizing, and capping agents for nitrate and chloride precursors during the green synthesis of MNPs [36,75]. Figure 3 schematically depicts the process of plant-mediated biosynthesis of MNPs.

The green synthesis of MNPs using plant extracts offers many benefits over other biological resources due to their wide availability, cultivability, and cost-effectiveness [76,77]. The optical, magnetic, and thermal characteristics of MNPs make them worthwhile candidates for use in various medical applications, including drug delivery, medical diagnostics, therapeutic goals, and so forth [78]. 

**Figure 4:**
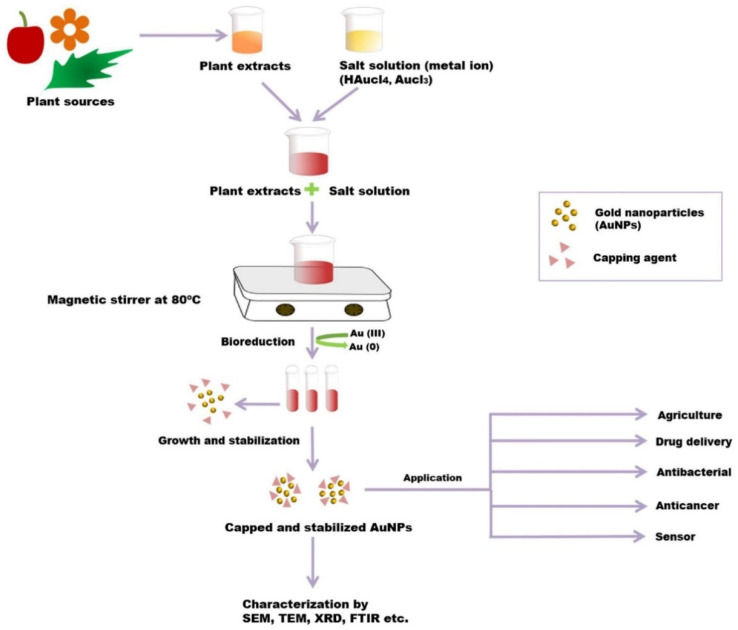
Schematic representation of the green synthesis of AuNPs using a plant. The characterized plant-based AuNPs have a variety of applications in agriculture and medicine (as nanosensors and drug delivery vehicles). Reprinted with permission from Ref. [82]. Copyright 2021 MDPI.

Various chemical and physical synthesis methods have been designed for AuNPs. Still, most of them are not accepted because of the use of toxic chemicals and the excessive heat produced in the synthesis process [79,80]. Extracellular NP synthesis is one of the most frequently used biosynthetic methods for fabricating AuNPs [81]. The green synthesis of AuNPs has been performed using plant tissues, microorganisms, actinomycetes, etc. (Figure 4).

## 5. Green Synthesis of AuNPs for Cancer Theranostics

The application of AuNPs is an exciting topic due to their distinctive surface plasmon resonance (SPR) and electrical conductivity [83], low toxicity [84,85], resistance to oxidation [86], their remarkable catalytic activity [87], and their biological potential for use in biomedical applications such as drug delivery systems [88], biosensing [89], molecular imaging, and diagnostic tools [90]. As a result, there is an increasing demand for eco-friendly biosynthesis procedures of AuNPs to ensure their safety and minimize unwanted consequences in biomedical applications. Indeed, the production of AuNPs using biological approaches is superior to physical and chemical methods due to their potentially increased biocompatibility.

**Figure 5:**
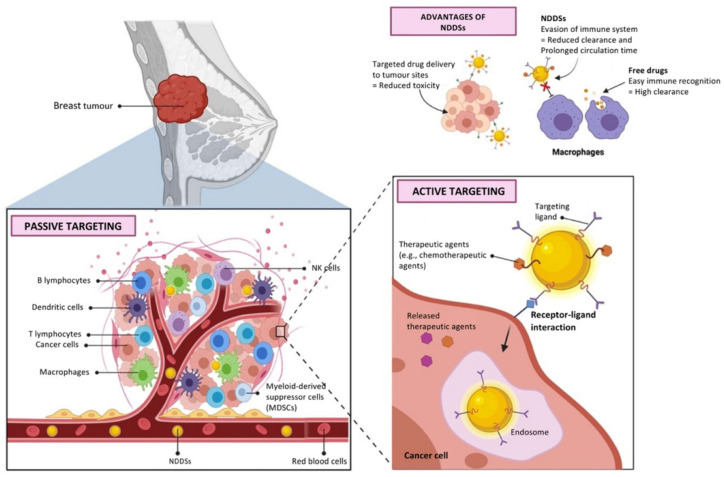
Schematic design for targeted drug delivery system procedure based on AuNPs. Reprinted with permission from Ref. [93]. Copyright 2021 Dove Press.

AuNPs can deliver a variety of medicines to the targeted tumor location without causing toxicity to the healthy or surrounding tissues of the tumoral site, including hydrophobic and hydrophilic pharmaceuticals, plant-based drugs, peptides, antibodies, short interfering ribonucleic acids (siRNAs), antibiotics, chemotherapeutic agents, and small-molecule medications [91,92]. The medicine enclosed in the AuNPs’ coating is protected against enzyme degeneration in the bloodstream [92]. Considering the therapeutic potential of plants in cancer therapy, the application of AuNPs as nanocarriers, especially biosynthesized AuNPs that are nontoxic and biocompatible for delivery of plant-based therapeutic biomolecules, could be a significant stride forward in cancer treatment without side effects. In addition, these nanocarriers can be an effective system where many drug moieties are surrounded by each AuNP and can enter the targeted cell. Figure 5 depicts the controlled and targeted drug delivery system based on AuNPs in the treatment of breast cancer [93].

In 2003, for the first time, Gardea-Torresdey et al. reported the biosynthesis of AuNPs using a plant (*Medicago sativa*) [94]. In this type of biosynthesis, the application of medicinal plants as reducing agents causes absorption of different biomolecules onto the metallic surface and constructs a biological halo circumference progressively. This circumference layer provides extra efficacy over nude biological NPs and offers potential biomedical applications, especially in antimicrobial and human therapy [95]. Simultaneously, because of their optical and chemical characteristics, AuNPs have great potential as diagnostic and therapeutic tools in various medical applications [96,97]. Nowadays, AuNP-based drug delivery systems with medicinal uses, such as AuNPs developed for cancer treatment and tumor targeting, have attracted significant attention [92]. The attachment of specific function-targeting ligands through surface chemistry on the AuNPs enables these NPs to act as potential carriers of anti-cancer agents [92].

### 5.1. Plant-Based AuNPs for Cancer Detection

**Figure 6:**
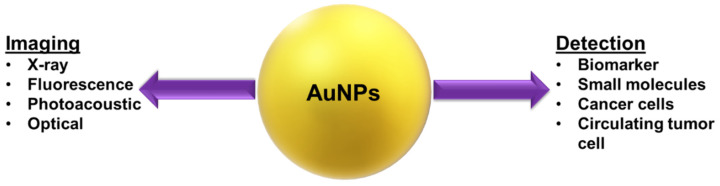
Application of AuNPs for cancer detection and imaging.

The high atomic number and electron density of Au lead to enhanced irradiation absorption at high X-ray tube voltages (120–140 kV) in comparison to iodine-based compounds [98,99]. AuNPs provide long-term imaging and monitoring of target cells since they have a longer circulation time, allow easy modification with ligands that are recognized by cancer cell surface receptors, and experience increased uptake through receptor-mediated endocytosis (RME) as compared to contrast agents currently used in the clinical field [100,101,102]. Considering these properties, studies have been carried out on the use of AuNPs as contrast agents for X-ray-based imaging techniques such as computed tomography (CT). The synthesis of nanoparticles (NPs) from plants, especially using cell-free plant extracts, is called green synthesis [103,104]. Green synthesized AuNPs are of great interest in cancer detection and diagnosis due to their advantageous properties, such as non-immunogenicity, high permeability, low cytotoxicity, and high stability [105]. The biocompatibility of green synthesized AuNPs is relatively high since they do not require chemical hazardous reducing, stabilizing, and capping agents, a crucial feature of biomedical applications. They also exhibit biological activity according to their synthesized shape and size [106]. Another critical issue is metastasis monitoring for cancer patients. Generally, AuNPs spread throughout the lymphatic system, allowing monitoring of metastases in patients with lymphatic system disease [107]. Green synthesized AuNPs, which are currently being used in targeted drug delivery, are now being applied in imaging diagnoses, such as X-ray imaging, fluorescence imaging, NIR imaging, and photoacoustic imaging, as they accumulate in the tumor at the cellular level [108]. Since the early diagnosis of cancer is vital, developing methods for this purpose is critical for reducing the devastating effects of this disease in the future and improving the survival time [109]. Chemically and green synthesized AuNPs can detect specific cell types of cancer or their biomarkers by using imaging and biosensor systems (Figure 6).

#### 5.1.1. Fluorescent-Plant-Based Markers for Detecting Cancers

Compounds naturally found in plants that have fluorescent properties can bind to NPs during green synthesis [110]. Fluorescent NPs synthesized with a green chemistry approach offer a low cost and less toxic properties in bioimaging than chemically synthesized NPs [111]. NPs modified with chemically synthesized fluorescent compounds are mostly used in imaging methods [112,113]. However, fluorescent NPs synthesized with chemical methods cause toxic effects due to the crosslinkers used during synthesis [103]. Furthermore, the addition of ligands is necessary to facilitate the targeted homogeneous distribution of the developed nanoparticle system within the body. Therefore, the green synthesis of NPs using plants rich in fluorescent compounds can overcome the limitations in bioimaging methods mentioned above. AuNPs to be synthesized by green chemistry can be obtained from plants showing fluorescence emission in the NIR region. Thus, NIR imaging can be successfully performed.

Examples of such fluorescent compounds obtained from plants are coumarin, caffeic acid, rhaponticin, alkaloid berberine and quercetin, rosmarinic acid, and ferulic acid. [114,115]. For example, Kotcherlakota et al. (2019) used the *Zinnia elegans* (ZE) plant to obtain green synthesized AuNPs. The reason for choosing the ZE plant in the study was that it shows fluorescence emission in the NIR region (red at 820 nm emission). The biocompatibility of the synthesized AuZE was tested with MTT in three different cancer cell lines (SK-OV-3, A549, and MCF-7), and its biocompatibility was proven. After intraperitoneal administration in C57BL6 mice, AuZE was found to be homogeneously distributed in the brain of mice without the need for ligands and exhibited bright red fluorescence in the NIR region (710 nm excitation, 820 nm emission). It has been stated that the green synthesized AuZE NPs developed in the study can be used in diagnostic imaging techniques [116].

Magnetic resonance imaging (MRI), one of the noninvasive diagnostic techniques, mostly uses superparamagnetic iron oxide nanoparticles (SPIONs) as a contrast agent. Whereas some studies have reported significant toxicity and ROS production in response to uptake of SPIONs by cells [117], others claimed that this toxicity is related to the surface coating, which affects the nanoparticle stability, aggregate size, and finally, cellular interaction [118,119]. For example, de la Fuente et al. reported that gold–iron nanoparticles coated with different saccharides influenced the responses of human fibroblast cells and their viability [120]. Production of hybrid NMs by combining different materials, such as iron oxide (Fe_3_O_4_) and green synthesized gold, can be used in MRI and CT for molecular diagnosis in cancer. For example, Narayanan et al. (2011) synthesized the Fe_3_O_4_/Au nanohybrid using grape seed proanthocyanidins (GSP) and used it as a contrast agent in MRI and CT imaging. The biocompatibility of the green synthesized Fe_3_O_4_/Au nanohybrids was found to be relatively high. It has also been reported that nanohybrids show superparamagnetism at high magnetic saturation, and the simple combination of gold with magnetite shows more efficient CT contrast than iodinated contrast agents currently in use in the clinical field [121].

Chanda et al. (2011) used cinnamon (Cin) to obtain green synthesized AuNPs to diagnose cancer cells. Normal human fibroblast (PC-3) and cancerous (MCF-7) cells were used to examine cytotoxicity and cellular uptake using a photoacoustic technique. Cin-AuNP biodistribution was tested on mice. In addition, a model of CT phantom was created. They observed that Cin-AuNPs were taken into cancer cells and produced very high photoacoustic signals. On the other hand, it was reported that Cin-AuNPs exhibited continuous accumulation in the lungs and rapid clearance from the blood in in vivo studies [122]. Mukherjee et al. (2012) used *Olax Scandens* (OX) leaf extract to synthesize AuNPs since OX has fluorescence, reducing, and stabilizing properties. They observed that synthesized AuNPs-OX displayed bright red fluorescence inside the lung (A549), breast (MCF-7), and colon (COLO 205) cancer cells compared to aqueous leaf extract. They reported that green synthesized AuNPs-OX could be used in both cancer diagnostics and therapeutics [123]. Morel et al. (2017) used *H. ambavilla* (leaves) and *H. lanceolatum* (flowering tops) plants to synthesize AuNPs. They used these plants as reducing agents to obtain green synthesized AuNPs and performed imaging of green AuNPs using a CT scan. According to their findings, green synthesized AuNPs exhibited nontoxic properties and can be used as multimodal imaging agents (CT scan and optoacoustic) to detect cancer cells [124]. *Barley leaf* (BL) extracts can be used for the green synthesis of AuNPs because of their strong reducing ability. In addition, it has been reported that AuNPs obtained from BL can be used to diagnose cancer [125]. NiChang et al. (2020) evaluated the CT imaging property of biosynthesized AuNPs using BL in vitro and a zebrafish model. The results also determined that it showed better biocompatibility and superior imaging properties than the traditionally used contrast agents in CT. It was stated that the BL-AuNPs developed in the study could be used in the imaging of cancer cells and biomedical applications [126].

#### 5.1.2. Other Plant-Based Markers for Detecting Cancers

Proteins or glycoproteins that are cancer biomarkers, such as prostate-specific antigen (PSA), carcinoembryonic antigen (CEA), cancer antigen 19-9 (CA 19-9), or mucin 16 (CA-125/MUC16), are relatively large molecules. However, exosomal, microRNAs, and circulating tumor nucleic acids (i.e., circulating tumor DNA (ctDNA), circulating tumor DNA (ctRNA), and circulating tumor cells are also used as cancer markers. Various biosensors have been developed using NMs for cancer diagnosis [127,128,129]. The usage of biosensors for diagnostic purposes in the clinical field increases daily. In particular, determining biological components as biomarkers in physiological fluids such as blood, urine, and saliva, called a liquid biopsy, is very important for diagnosing various cancers or other diseases [130]. Among these sensor systems, nanoparticle-based electrochemical sensors have grabbed attention with their low-cost, simple-to-use, and point-of-care analysis features [131]. Modifying the electrode surface with nanosized materials that possess high electrical conductivity makes it possible to obtain a low detection limit. In this context, electrochemical sensors offer excellent performance for the trace detection of biomolecules. AuNPs with an excellent catalytic effect and conductivity increases the performance of the sensor system to be developed [132]. Another material with very high electrical conductivity is reduced graphene oxide (rGO) and its derivatives. High-performance nanocomposite production can be achieved by combining both NMs [133]. Bringing these two materials together can be carried out indirectly or directly. The method of direct deposition of AuNPs on the surface of rGO is accomplished by reducing chloroauric acid on the surface of rGO. In the indirect deposition method, a covalent bond is formed between AuNPs and GO [134]. Nazarpour et al. (2020) developed an electrochemical sensor based on a green synthesized AuNPs/rGO nanocomposite to detect tryptophan, which is used as a biomarker for lung cancer. They carried out this green synthesis by using an *E. tereticornis* solution (extracted from the *Eucalyptus tree*) as a reducing agent. The linear detection range was found to be between 0.5 and 500 μmol/L (R = 0.9976), and the limit of detection (LOD) was calculated as 0.39 μmol/L. They indicated that the developed electrochemical sensor system is suitable for the determination of tryptophan in human plasma, serum, and saliva [135].

### 5.2. Plant-Based AuNPs for Cancer Treatment

**Figure 7:**
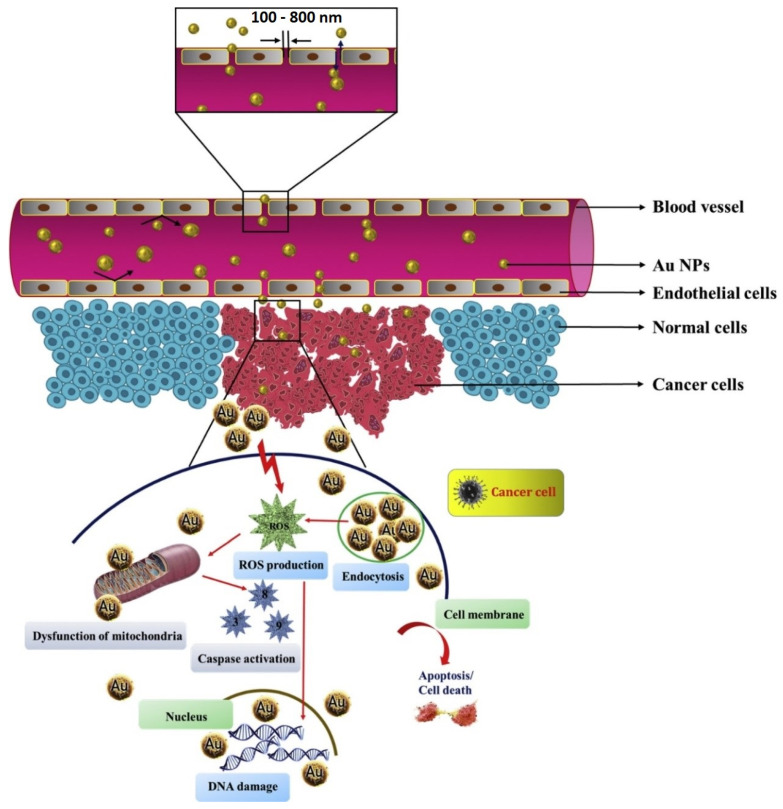
Suggested anticancer mechanisms of AuNPs. Reprinted with permission from Ref. [141]. Copyright 2021 Elsevier.

Responsible for one in seven deaths worldwide, cancer is continuing to claim the lives of millions of people [136]. According to an estimation by WHO, cancer will claim around 12 million lives by 2030 [137]. Conventional treatment strategies come with numerous side effects and present a major obstacle in effective cancer therapy [138,139]. With continuous research in nanotechnology, scientists strive to develop unique strategies for treating cancer with minimal side effects and an improved survival rate. Metallic NPs have been reported in the literature for their cytotoxic effect against cancer cells. More recently, green synthesized AuNPs have gained considerable attention in this regard. The cytotoxicity of metal NPs primarily depends on the particle size, surface reactivity, and surface area [136]. In addition, the characteristics of plant extracts have a key role in the internalization of AuNPs along with synergistic cytotoxic effects [137]. Various mechanisms are responsible for the anticancer activity of AuNPs (Figure 7). For example, (a) alteration in the cell permeability leading to mitochondrial dysfunction [140], (b) generation of ROS causes oxidation stress and fragments DNA, finally activating apoptotic pathways that can be caspase-dependent or independent, (c) alteration in the chemical structure of proteins/DNA, and (d) cell cycle arrest [82]. This section highlights various research works carried out in recent years to investigate the anticancer effect of various green synthesized AuNPs towards different cancer cell lines.

#### 5.2.1. Breast Cancer

Breast cancer, the most frequent type of cancer in women, is the second most prominent cause of death related to cancers, with nearly 25% cases of high prevalence in developed countries [142]. An exciting research work employing four different green synthesized AuNPs in combination against breast cancer was carried out by Vemuri and fellow researchers. Curcumin, turmeric, quercetin, and paclitaxel were used to synthesize AuNPs, which were then characterized via different techniques. They then evaluated the cytotoxic effect of the individual AuNPs and combinations of AuNPs against MCF-7 and MDA-MB 231 cancer cell lines. Interestingly, anticancer activity was significant when combining all four biosynthesized AuNPs compared to their free forms. The combination of AuNPs synergistically inhibited cell proliferation, angiogenesis, spheroid formation, and colony formation. However, neither combination form nor individual forms demonstrated any cytotoxicity against the human embryonic normal kidney cell line (HEK 293), suggesting the biocompatibility of biosynthesized AuNPs [142].

Green synthesized AuNPs have been tested mostly against MCF-7 cancer cell line, and various ways in which AuNPs have interacted with MCF-7 cells have been reported, including alteration of the integrity of cell membranes, oxidative stress, interrupting physiological as well as metabolic processes, severing synthesis of ATP, halting the transfer of electrons, and finally, shrinking of cells and apoptosis [143,144,145]. Results of the study conducted by Devendiran et al. [146] unveil the prodigious potential of AuNPs as carriers of chemotherapy drugs in drug delivery systems. Doxorubicin (DOX) loaded onto pectin-derived AuNPs (pec-AuNPs) showed a significant cytotoxicity effect against breast cancer cells compared to free DOX. Additionally, they reported that AuNPs decorated with chitosan-folic acid could be considered biocompatible nanocarriers for targeted drug delivery towards cancer.

Hoshyar et al. reported the one-step synthesis of AuNPs using crocin as a reducing agent. Crocin was obtained from the *saffron stigma*, which contains many active phytocompounds. Crocin is an antioxidant that causes the reduction of free radicals in order to protect cells from oxidative stress. The researchers also tested the anti-proliferative effect of crocin-AuNPs on human breast cancer cells via an MTT assay and neutral red test. An LDH (lactate dehydrogenase) test was carried out to measure the release of LDH enzymes from the cytoplasm. The IC50 values of crocin-AuNPs were 1.8 mg/mL ± 0.08 and 1.2 mg/mL ± 0.04 after incubating for 24 and 48 h, respectively. Results from all tests indicate significant inhibition of proliferation of cancer cells in a dose- and time-dependent manner. The researchers attributed these observations to the enhanced entry of synthesized crocin-AuNPs into the cancer cells followed by the release of conjugated crocin from AuNPs [147].

Vinayagam et al. used an aqueous extract of *Cynodon dactylon* (Bermuda grass) for the green synthesis of AuNPs. The MCF-7 breast cancer cell line and NIH 3T3 noncancerous cell line were used to study the cytotoxicity of synthesized AuNPs. Even at higher concentrations, no significant cytotoxicity was observed in the NIH 3T3 cell line. In the case of MCF-7 cancer cells, the inhibitory effect was found to be dose-dependent, and the IC50 value of green synthesized AuNPs was shown to be 31.34 µg/mL. Upon treating the MCF-7 cancer cells with an IC50 concentration of C.dactylon-AuNPs, the ROS levels increased, leading to programmed cell death. Results of rhodamine staining, AO/EtBr, and DAPI staining show notable mitochondrial membrane damage and DNA fragmentation, suggesting apoptosis in cancer cell lines [148]. Another study carried out by Uzma and fellow researchers showed the potential of green synthesized AuNPs against breast cancer. The aqueous extract of *Commiphora wightii* was utilized for fabricating AuNPs. C.wightii, traditionally known as the *Guggul* tree, is known for its various medicinal applications, including anticancer characteristics. In order to investigate the cytotoxic behavior of Cw@AuNPs, the MCF-7 cell line was used. Various in vitro cytotoxicity tests were performed, including a comet assay, flow cytometry, DNA fragmentation, and EtBr dual staining. A concentration-dependent inhibitory effect was observed with an IC50 of 66.11 μg/mL. The enhanced cytotoxic effect of AuNPs can be attributed to the existence of secondary metabolites in the medium used for synthesizing the AuNPs. Cell cycle analysis revealed cell cycle arrest at the G2M phase that consequently induced apoptosis [149].

Li et al. used *Mentha Longifolia* leaf extract for the environmentally friendly synthesis of AuNPs and conducted cytotoxicity studies on common breast cancer cell lines (breast carcinoma (Hs 578Bst), breast adenocarcinoma (MCF7), breast-infiltrating lobular carcinoma (UACC-3133), and breast-infiltrating ductal carcinoma cells (Hs 319.T). The green synthesized AuNPs exhibited dose-dependent anticancer activity against all four cancer cell lines, with the most significant effect on the UACC-3133 cell line. The IC50 values of AuNPs were found to be 274 µg/mL against MCF-7 cells, 279 µg/mL against the Hs 578Bst cell line, 274 µg/mL against the Hs 319.T cell line, and 201 µg/mL against the UACC-3133 cell line [150]. Similar results were reported by Kiran et al., who used an extract of *Moringa oliefera* leaves as a bioreductant for synthesizing AuNPs. The MCF-7 cancer cell line was used to study the cytotoxic effect of MO-AuNPs. An IC50 value of 67.92 μg/mL was observed to induce the concentration-dependent anticancer activity of MO-AuNPs [151].

A comparative study was carried out by Hosny et al. using two halophytic species (*Atriplex halimus* and *Chenopodium amperosidies*). AuNPs were prepared in a single-step method, and the anticancer efficacies of *A. halimus*-AuNPs and *C. amperosidies*-AuNPs were investigated in the MCF-7 cancer cell line via an MTT assay. It was observed that the efficiency of *A. halimus*-AuNPs (IC50 = 47.03 μg/mL) in inhibiting the growth of cancer cells was higher than that of *C. amperosidies*-AuNPs (IC50 = 22 μg/mL) [152]. AuNPs synthesized using dragon fruit extract showed significant anti-proliferative activity against MCF-7 and MDA-MB-231 cells using an Alamar blue assay. Due to their extremely small size and high surface area, the AuNPs penetrated the tumor sites. In the case of MCF-7 cells, maximum inhibition of around 80% was achieved at 500 µg/mL after an exposure time of 48 h. However, no substantial toxicity was observed in MDA-MB-231 cells. The researchers attributed the sensitivity of MCF-7 cells to the DF extract and that of DF-AuNPs to the presence of hormone receptors and low metastatic potential, whereas MDA-MB-231 cells lacked these receptors and hence showed low sensitivity [153].

Suganya et al. documented time- and dose-dependent anticancer activity with green synthesized AuNPs using the leaf extract of *Mimosa pudica* against breast cancer cell lines (MCF-7 and MDA-MB-231). Alterations in cancerous cells, such as cell shrinkage, changes in membrane integrity, inhibition of cell growth, and nuclear condensation, were observed in both cell lines upon treatment with synthesized AuNPs. Cell cycle analysis demonstrated significant apoptosis in the early G0/G1 to S phase. After an exposure time of 48 h, the IC50 value was found to be 4 µg/mL in the case of MDA MB231 cells, whereas it was 6 µg/mL in the case of MCF-7 cells. The cytotoxicity was suggested to be time-dependent since 50% inhibition was not achieved after exposing both cell lines for 24 h. Results of the comet assay, propidium iodide (PI), and 4′,6-diamidino-2-phenylindole (DAPI) staining reveal DNA damage in treated cells compared to control cells [154]. 

#### 5.2.2. Cervical Cancer

Cervical cancer is known to be the third-highest cause of mortality in women globally. Human papillomavirus is associated with 99% of cases [155]. HPV-positive cervical cancer cells tend to resist chemotherapy-induced cell death and suppression of cell proliferation [156]. Ke et al. studied the cytotoxic potential of AuNPs prepared using a leaf extract of *Catharanthus roseus* against the HeLa cancer cell line. Catharanthus roseus is a medicinal plant that is the main source of vincristine and vinblastine. MTT assay was used to evaluate the IC50 value of synthesized AuNPs, which was found to be 5 μg/mL. Further testing suggested that CR-AuNPs stimulated cell death in HeLa cells through ROS-mediated mitochondrial pathways, leading to apoptosis. Additionally, CR-AuNPs remarkably enhanced the activity of caspase-3 and caspase-9 and the expression of pro-apoptotic proteins (Bax and Bid), which suggests apoptosis in HeLa cells [157].

Similarly, Qian et al. reported the green synthesis of AuNPs using an aqueous extract of *Alternanthera sessilis* [155]. *A. sessilis*, commonly known as joy weed, belongs to the family of *Amaranthaceae* and demonstrates several therapeutic as well as pharmacological effects. Cytotoxicity of synthesized AuNPs on HeLa cells was assessed using an MTT assay, which demonstrated a concentration-dependent effect (10–15 μg/mL) on inhibition of cervical cancer cells. Furthermore, it was observed that Bax expression was upregulated, whereas expression of Bcl-2 and Bid was downregulated in HeLa cells. The activity of caspase8, 9, and 3 was found to be increased in HeLa cells in a dose-dependent manner; hence, induction of apoptosis occurred in cancer cells through intrinsic pathways [155].

Baharara et al. synthesized a leaf extract of *Zataria multiflora* for fabricating AuNPs and documented caspase 3 and 9 activation that induced apoptosis in HeLa cells [158]. Kajani et al. fabricated colloidal AuNPs using aqueous and ethanolic extracts of *Taxus baccata* and tested their anticancer activity against three prevalent cancer cell lines, including MCF-7, Caov-4, and HeLa. T.Baccata contains diterpenoid taxanes in large amounts, which are known to impart stabilizing properties to metal nanoparticles and deliver new biological features such as anticancer activity to NPs [159]. Various tests were carried out that demonstrated the high toxicity potential of the synthesized biogenic AuNPs against all three cancer cell lines. It was observed that AuNPs synthesized using ethanolic extracts exhibited significantly higher cytotoxicity against all cell lines compared to those prepared using aqueous extracts. In the case of HeLa cells, the cell mortality was found to be 98% and 84% using ethanolic and aqueous extracts synthesized AuNPs, respectively. The authors suggested the contribution of organic compounds of *Taxus* extracts that were absorbed on the surface of AuNPs to an enhanced anticancer effect. A time- and dose-mediated cell death involving a combination of necrosis and apoptosis was seen in AuNP-treated cancer cells. Furthermore, the transcription level of Bcl-2 changed in a dose-dependent manner in treated cells, whereas the expression of caspase 8 and 9 remained significantly unchanged. Overall, results suggest caspase-independent cell death as a possible mechanism of anticancer activity of synthesized biogenic AuNPs [159].

#### 5.2.3. Liver Cancer

Hepatocellular carcinoma (HCC) or liver cancer, another malignancy with a high mortality rate, is found to have been stabilized in men, whereas it is on a steady rise in women owing to various factors, as reported by Global Cancer Statistics 2021 [160]. Surgical resection is the most common treatment option for liver cancer; however, late diagnosis in patients makes surgical resection unfeasible [161].

Rajeshkumar et al. tested the cytotoxicity of AuNPs against liver cancer in vitro. A green synthesis approach was adopted using fucoidan, a sulfated polysaccharide. Brown seaweed is considered the primary source of fucoidan. Owing to its antioxidant, antiviral, antithrombotic, and antitumor properties, fucoidan has several applications in the field of medicine [162]. In this study, fucoidan acted as a reducing and stabilizing agent. The anticancer activity of synthesized AuNPs was then assessed against the HepG-2 cell line via an MTT assay. Various concentrations of green synthesized AuNPs were employed to investigate the anticancer activity. Results show that minimum inhibitory activity occurred at 10 µg, whereas high inhibitory action was achieved at 100 µg, suggesting the concentration-dependent effect of AuNPs on inhibiting proliferation in HepG2 cancer cells. The researchers attributed the inhibitory action of AuNPs to the nanoparticle size (31 nm), which induced oxidative stress and reduced the cell viability, resulting in apoptosis [162]. Researchers from China chose *Cordyceps Militaris*, a mushroom species that had been used as a traditional Chinese medicine for centuries. AuNPs were fabricated and characterized through various techniques. Assessment of cytotoxicity was carried out on the HepG2 cell line, and the 50% inhibitory concentration was found to be between 10 µg and 12.5 µg/mL. Activation of Bax, Bid, and caspases and inhibition of bcl-2 activation was suggested as possible mechanisms of apoptosis. ROS is generated, which significantly damages the membrane potential of mitochondria in HepG2 cancer cells [163].

The seed extract of *Trachyspermum ammi* was used by Perveen et al. to prepare AuNPs through a rapid-microwave-assisted approach. Upon examining the anticancer activity of biosynthesized AuNPs against the HepG2 cell line, it was noticed that TA-AuNPs inhibited the cancer cells’ proliferation in a time- and dose-dependent manner. The IC50 value was observed to be 92.453 µg/mL, and the HepG2 cells responded to TA-AuNPs within 48 h. Further investigation revealed that increasing TA-AuNPs’ concentration resulted in an increase in lipid peroxidation in HepG2 cells. Lipid peroxidation is induced due to ROS generation, which has a significant role in cell death. In addition, TA-AuNPs also depleted GSH levels in HepG2 cancer cells, where a 75% reduction in GSH levels was observed at 200 µg/mL of TA-AuNPs, which further manifests the potential of TA-AuNPs as an effective anticancer therapy [164]. Peel and pulp extract of *Annona muricata*, commonly known as Graviola, were employed in a study for fabricating AuNPs. The synthesized AuNPs showed no toxicity against normal VERO cells, whereas significant cytotoxicity against Hep2 cells was observed with an IC50 value of 10.94 μg/mL. The anticancer activity of peel extract synthesized AuNPs was found to be higher when compared with those prepared using pulp extract [165].

*Dendrobium officinale* (DO) synthesized AuNPs have shown significant cytotoxicity against cancerous liver cells in vitro and in vivo. Results of the CCK8 assay, a nonradioactive, sensitive colorimetric assay that allows precise live-cell counting in cell proliferation or cytotoxicity assay applications, confirm the inhibitory effect of green synthesized AuNPs on HepG2 cells and exhibited a protective effect on L02 cells. A reduction in tumor volume and body weight was observed along with an inhibited tumor growth rate when Do-AuNPs were tested in vivo. Results of Ki-67 and TUNEL assays further confirm the cytotoxic effect of Do-AuNPs on the tumor. Serum levels of aspartate aminotransferase (AST), creatinine, alanine aminotransferase (ALT), and blood urea nitrogen (BUN) of mice along with hematoxylin and eosin staining of the liver, lung, heart, kidney, and spleen established the biosafety of AuNPs [161].

#### 5.2.4. Colon Cancer

Ranked third among cancers for the highest number of deaths, colon cancer is treated using traditional approaches, including chemotherapy, surgery, and radiation [166]. However, these therapies’ limitations and side effects call for advanced strategies for improving treatment efficacy with minimal toxic effects [167]. Researchers from Spain presented green synthesized AuNPs through a fast, one-pot technique using an aqueous extract of *Cystoseira baccata*, brown macroalgae, and further investigated their cytotoxicity towards colon cancer cell lines (Caco-2 and HT-29). Overall, it was observed that the cytotoxic effect was stronger against HT-29 than Caco-2 cancer cells. However, no toxicity was observed upon testing against the normal neonatal dermal fibroblast cell line (PCS-201-010). When the apoptotic activity was investigated, it was shown that Au@CB induced apoptosis by activating extrinsic and mitochondrial pathways in vitro. The IC50 values were reported to be 79.03 µM and 49.61 µM for Caco-2 and HT-29, respectively. Flow cytometric analysis was conducted to check the redistribution of phospholipid phosphatidylserine (PS) and membrane permeabilization [168].

In a similar study, Hosseinzadeh et al. synthesized AuNPs using essential oil of *Ferula persica* gum and investigated them in vitro anticancer effects against the CT26 cell line. Dose-dependent cytotoxic activity was observed with an IC50 value of 0.0024 mg/mL against CT26 cells. This value was found to be 0.0307 mg/mL when tested against normal Vero cell lines. Hence, the Au NPs induced more pronounced apoptosis against the colon cancer cell line than the normal Vero cells. The clonogenic assay revealed inhibition of colony formation in both cell lines when compared to untreated cells [169]. Abel et al. investigated the anticancer activity of AuNPs synthesized using *Cassia tora* against colon cancer cells. *C.tora* was introduced as a capping agent and as a bioreductant. A reduction in cell viability of Col320 cancer cells was observed with an MTT assay. The release of markers, including nitric oxide, lipid peroxidase, and hydrogen peroxide, was also suppressed to a great extent in a dose-dependent way. In addition, catalase was measured, and results show increased catalase activity upon increasing the concentration of C.tora-AuNPs. This leads to the breakdown of hydrogen peroxide in cancer cells [170]. An IC50 value of 48 μg/mL was achieved against HCT-116 cells employing AuNPs synthesized using a leaf extract of *Albizia lebbeck (AL)*. Apoptosis was induced due to ROS generation, reduced mitochondrial membrane potential, and expression of pro- and anti-apoptotic proteins [171]. *Allium sativum* has therapeutic potential against various types of cancer. Liu et al. fabricated AuNPs using an aqueous leaf extract of *A. sativum* and examined their anticancer activity against various colon cancer cells using an MTT assay. The 50% inhibitory concentrations were achieved at 269 μg/mL against HT-29 cells, 225 μg/mL against HCT 116 cells, 250 μg/mL against HCT-8 [HRT-18] cells, and 236 μg/mL against Ramos.2G6.4C10 cells. The AuNPs exhibited their cytotoxic effect in a dose-dependent manner. However, the most significant effect was observed in the case of HCT 116 cells [172].

Dakhtile and fellow researchers employed the HCT-15 cancer cell line to examine the anticancer activity of AuNPs synthesized via a green approach using an aqueous extract of *Argemone Mexicana*. MTT and DNA fragmentation assays were conducted to evaluate cytotoxicity and genotoxicity, respectively. RT-PCR was employed to measure the expression of apoptotic proteins, whereas Western blot analysis was carried out to confirm cell death in cancer cells. Results show IC50 values of 20.53 μg/mL and 12.03 μg/mL after exposing the cells to biogenic AuNPs for 24 and 48 h, respectively. Increased apoptosis with altered morphology of cells was observed together with overexpressed p53 and caspase-3 [173].

Spherical AuNPs with a 25 nm particle size were developed using *Prosopis farcta* extract, which has the ability to reduce Au^+3^ to Au^0^ due to the presence of certain phenolic compounds. *P. farcata* is a medicinal herb indigenous to the Middle East. An MTT assay was carried out to study the cytotoxic effect of biosynthesized AuNPs against the HT-29 cell line, and 50% inhibition was achieved at 419.7 µg/mL. Further, the TUNEL assay was applied to examine the apoptotic activity of synthesized AuNPs at various concentrations. The apoptotic activity was found to increase when the concentration of AuNPs was increased up to 200 µg/mL; however, no significant cell death in HT-29 cells was observed beyond this concentration. The researchers also attributed the apoptotic effect to changes in cell morphology, mitochondrial damage, and oxidative stress [174].

Earlier, Mata et al. also reported a similar anticancer mechanism of AuNPs synthesized using a leaf extract of *Abutilon indicum*. FTIR and GC-MS studies confirmed the existence of polyphenolic groups, which were thought to stabilize the AuNPs. The anticancer activity was observed in HT-29 cells with reported IC50 values of 210 µg/mL after 24 h of exposure and 180 µg/mL after 48 h of exposure. AO/EtBr, PI, and AnnexinV-Cy3 staining techniques were employed for studying the anticancer mechanism. Both intrinsic and extrinsic apoptotic pathways were prompted by ROS generation, depletion of cellular antioxidants, and increased expression levels of caspase-3, caspase-8, caspase-9, PARP, and Lamin A/C [175]. AuNPs synthesized using *Ganoderma lucidum* exerted a cytotoxic effect against HT-29 cells in a dose-dependent manner. The IC50 value was calculated to be 84.58 µg/mL. *G.lucidum* is a mushroom that is rich in polyphenols, turpentines, and flavonoids. The resultant AuNPs were of different shapes with a size range of 1–100 nm [176].

#### 5.2.5. Lung Cancer

Several studies have also been carried out to study green synthesized AuNPs in lung cancer treatment. Lung cancer can be broadly divided into two types, small cell lung cancer (SCLC) and nonsmall cell lung cancer (NSCLC) [177]. Extract of *Marsdenia tenacissima* was used as a stabilizing agent by Sun et al. for synthesizing AuNPs in order to test their anticancer activity against the A549 lung cancer cell line. Dose-dependent cytotoxic activity of AuNPs was determined with an MTT assay with an IC50 value of 15 µg/mL. The authors also suggested the contribution of active molecules in the extract of *M. tenacissima* adsorbed onto AuNPs for enhancing anticancer activity. Further testing showed down-regulation of anti-apoptotic proteins and upregulation of caspases in A549 cells [178]. Vijayakumar et al. employed a peel extract of *Musa paradisiaca* to fabricate AuNPs facilely. The fabricated MPPE-AuNPs were further tested for their anticancer activity in A549 cells using various concentrations. The IC50 value was calculated to be 58 µg/mL. Increasing the concentration of MPPE-AuNPs to 100 µg/mL caused the cell viability to decrease, suggesting the effective inhibition of lung cancer cells. At 100 µg/mL, the biosynthesized AuNPs induced morphological changes in A549 cells after an exposure of 24 h [179].

Anand et al. [180] reported the influential role of *Moringa oleifera* flowers in reducing Au ions and producing AuNPs. Their assessment revealed that biosynthesized AuNPs were toxic against A549 lung cells, whereas they did not exhibit a cytotoxicity effect on healthy peripheral blood mononuclear cells (PBMCs). Tiloke and fellow researchers also documented the anticancer activity of *Moringa oleifera*-AuNPs against A549 cells. Their results suggest that ML-AuNPs were cytotoxic to A549 cells, whereas in healthy PBMCs, no significant cytotoxicity was seen. In A549 cells, cytotoxicity was manifested through the intrinsic apoptotic pathway. The ML-AuNPs target tumor suppressor genes and oncogenes and activate alternate splicing of caspase-9 to induce apoptosis effectively in A549 cells. Further testing against SNO oesophageal cancer cells showed a decrease in cell viability in a dose-dependent manner along with activation of caspase activity, which emphasized the potential anticancer activity of ML-AuNPs against cancer cells. Upon comparing ML-AuNPs with chemically synthesized tri-sodium citrate AuNPs, it was found that green synthesized ML-AuNPs not only significantly diminished the viability in A549 cells but also stimulated higher caspase activity in SNO cells [181].

Recently, El-borady et al. validated the anticancer activity of green synthesized AuNPs against A549 lung cancer cells. They used the aqueous leaf extract of *Phragmites australis*, also known as common reed, to synthesize the AuNPs and further characterize them using UV–Vis spectroscopy, FTIR, high-resolution transmission electron microscopy (HRTEM) mapping, X-ray diffraction (XRD), energy-dispersive X-ray analysis (EDX), X-ray photoelectron spectroscopy (XPS), and zeta potential. The MTT assay showed an IC50 value of 129 μg/mL with significant anticancer activity against A549 cells [182].

#### 5.2.6. Hematological Malignancies

Biosynthesized AuNPs have also been examined for anticancer activity against leukemia, a type of blood cancer. For example, the aqueous leaf extract of *Centaurea behen* was used to fabricate ecofriendly AuNPs, which were then tested for cytotoxicity and antioxidant activity against the leukemia cell line (THP-1) using an MTT assay and DPPH test. The biosynthesized AuNPs showed cytotoxicity against THP-1 cells with an IC50 value of 25 µg/mL. An increase in the concentration of AuNPs resulted in increased antioxidant activity [183]. Similar results were reported by Zhao et al., who applied *Tribulus terrestris* extract as a stabilizer in the fabrication of AuNPs and tested them against the THP-1 cell line for evaluating the antioxidant and anticancer activity. The 50% inhibition of cancer cells was achieved at 468 µg/mL. In addition, staining assays were conducted, and necrosis was observed as a cause of cell death [184].

Chen et al. studied the antileukemia and antioxidant properties of AuNPs synthesized using aqueous leaf extract of *Cannabis sativa*. Acute T-cell leukemia and lymphoblastic leukemia cell lines were used. A dose-dependent decrease in cell viability was observed in MOLT-3 (IC50 = 329 μg/mL) and TALL-104 (IC50 = 381 μg/mL) cell lines. A similar effect was observed in T-cell leukemia cell lines Jurkat, Clone E6-1 (IC50 = 502 μg/mL), and J.RT3-T3.5 (IC50 = 567 μg/mL). No cytotoxicity was observed in the case of a normal HUVEC cell line. The antioxidant potential was measured, and the authors suggested a relationship between the antileukemia activity of biosynthesized AuNPs and their antioxidant potential [185].

In another in vivo investigation, Ahmeda et al. reported the green synthesis and chemical characterization of AuNPs synthesized using *Camellia sinensis* leaf aqueous extract to treat acute myeloid leukemia in a leukemic mouse model. The fabricated AuNPs were then characterized via several techniques, such as UV-Vis., FTIR spectroscopy, TEM, EDS, FE-SEM, and XRD. The morphology of the developed nanoformulation was found to be spherical with diameters between 20 and 30 nm. The nanoformulation improved anti-inflammatory cytokines, lymphocytes, platelets, and red blood cell (RBC) indices and lowered the mass and size of livers and spleens while decreasing pro-inflammatory cytokines, to the same extent as daunorubicin, a chemotherapeutic agent used to treat hematological malignancies [186].

Likewise, in a rodent model, Zanganeh et al. used Hibiscus sabdariffa flower extract to fabricate novel AuNPs for leukemia treatment. The same techniques, including TEM, FESEM, XRD, FTIR, and UV-Vis, were applied to characterize the size of developed AuNPs, and their size was found to be between 15 and 45 nm, with a spherical shape. Similar to daunorubicin, plant-mediated AuNPs diminished the inflammatory cytokines (IL1, IL6, IL12, IL18, IFNY, and TNFα), total WBC, blast, monocyte, neutrophil, eosinophil, and basophil counts, and increased RBC indices and IL4, IL5, IL10, IL13, and IFNα levels. The nanoformulation exerted low cytotoxic effects against normal human (HL-60/vcr and 32D-FLT3-ITD) and murine (C1498) cells and exhibited no toxicity against HUVECs [187]. These observations provide a rationale for the in vivo application of plant-mediated AuNPs in cancer therapy

#### 5.2.7. Other Cancers

Researchers are also making efforts to study the effect of green synthesized AuNPs on other cancers. For example, Chen et al. recently reported the anticancer potential of green synthesized AuNPs against human ovarian cancer cell lines (SW-626, SK-OV-3, and PA-1). The aqueous leaf extract of *Curcumae Kwangsiensis* was used to synthesize AuNPs. An MTT assay was carried out to investigate and compare the cytotoxicity of pure leaf extract, gold chloride, and biosynthesized AuNPs. The AuNPs exhibited the best results with low cell viability and dose-dependent high anticancer activity in PA-1, SW-626, and SK-OV-3 cell lines. However, the AuNPs notably showed no cytotoxicity against the HUVEC normal cell line. Researchers reported the IC50 values of AuNPs to be 204 mg/mL, 166 mg/mL, and 153 mg/mL against SK-OV-3, SW-626, and PA-1, respectively. In addition, a DPPH assay was conducted to study the antioxidant potential of all three substances. Again, the results show the higher antioxidant activity of AuNPs. The researchers attributed the high cancer activity of AuNPs to their antioxidant effects [188].

In 2019, research on the AuNPs biosynthesized using *Scutellaria barbata* displayed prodigious potential against pancreatic cell lines [189]. Wang and fellow researchers tested green synthesized AuNPs against pancreatic cancer cells (PANC-1) using the extract of *Scutellaria barbata*. They also observed time- and dose-dependent anticancer activity of AuNPs against cancer cells. To further study the cytotoxic effect and determine cell death mode in cancer cells, a fluorescent staining method was employed using 25 µg/mL and 50 µg/mL of synthesized AuNPs. Acridine orange (AO) and PI staining showed condensed chromatin and blebbing of cancer cell membranes, indicating apoptotic activity of AuNPs. Generation of ROS, slight downregulation of Bcl-2, and upregulation of Bax, caspase-9, and caspase-3 proteins were suggested to be the possible mechanisms of apoptosis [189].

Researchers from India put forth the cytotoxic potential of green synthesized AuNPs against prostate cancer cell line (PC-3). Hydrothermal synthesis technique was employed for fabricating NPs using seed extract of *Elaeocarpus ganitrus*. XRD and TEM techniques were performed for the characterization of AuNPs. In addition, the anticancer and antioxidant activity of the AuNPs were studied using an MTT assay and DPPH assay, respectively. The in vitro investigation revealed a dose-dependent cytotoxic activity of AuNPs against the PC-3 cell line. The IC50 value was reported to be 64.23 µg/mL [141].

In vitro testing of *Curcuma wenyujin*-based AuNPs against renal cancer was studied by a group of researchers from China. A498 and SW-156 renal cancer cell lines were chosen for this purpose. An MTT assay was carried out to measure cytotoxicity, whereas various staining techniques were applied to study the mechanism of apoptosis. Out of the two cell lines, A498 cells were found to be more sensitive towards CW-AuNPs. The obtained CC50 value for the A498 cell line was 25 µg/mL, whereas it was 40 µg/mL in the case of the SW-156 cell line. Apoptotic staining, along with the results of qPCR and immunoblotting assay, show upregulation of apoptotic caspase-3, caspase-9, Bad, and Bid, and downregulation of anti-apoptotic proteins Bcl-2 and Bcl-xl. Together, the results imply effective induction of apoptosis in renal cell lines using CW-AuNPs [190].

The effect of *Siberian ginseng*-based spherical AuNPs on skin cancer cells was examined by Wu et al. The aqueous extract of *S.ginseng* was used for fabricating the AuNPs. The SG-GNPs were further characterized using various techniques. The anticancer efficacy of SG-GNPs was then tested in murine melanoma cells (B16). The viability of melanoma cells decreased dose-dependently upon treatment with SG-GNPs, and the CC50 dose was 10 µg/mL. The staining technique demonstrated ROS generation and increased the mitochondrial membrane permeability of cells, resulting in the release of pro-apoptotic proteins. Finally, the gene expression of apoptotic proteins was upregulated, and the anti-apoptotic gene expression was downregulated. The results signify the potential of SG-GNPs as a potential candidate in inducing apoptosis in cancer cells [191].

Patil et al. prepared spherical and oval-shaped AuNPs with diameters in the range of 10–30 nm using the leaf extract of *Sasa borealis*. The cytotoxicity of biosynthesized AuNPs was then tested against normal HEK293 cells and gastric cancer cells (AGS) using a WST-1 assay. No effect on cell viability was observed in HEK293 cells using different concentrations of AuNPs (0–300 μg/mL). A time- and concentration-dependent effect was seen in AGS cells using 50, 100, 150, and 200 μg/mL of AuNPs. The IC50 value was achieved at 120 μg/mL. With further testing, researchers concluded ROS generation, cell cycle arrest, interruption in cell permeability, and activation of apoptosis mediated by caspase cascade were reasons for cell growth inhibition [192]. In 2020, Yun et al. also explored the anticancer activity of *Vitex negundo* AuNPs against gastric cancer and reported an almost similar mechanism for activation of apoptosis [193].

In an interesting study, researchers from Brazil employed *Brazilian red propolis (BRP)* extract and its fractions to synthesize AuNPs and further compared their anticancer activity in urologic cancer cell lines. BRP is a product obtained from bees and is globally known for its various therapeutic applications. The anti-proliferative and antitumor properties of BRP have been studied in in vitro and in vivo models (23–25). In one study, AuNPs were prepared from the extract of BRP (AuNP extract), and its fractions (AuNP hexane, AuNP dichloromethane, AuNPethyl acetate), and they in vitro cytotoxicity were evaluated in bladder cancer cells (T24) and prostate cancer cells (PC-3) using a resazurin assay. All samples showed dose-dependent cytotoxicity towards both cell lines; however, green AuNPs presented the highest cytotoxicity as compared to other nanoparticle samples. The IC50 value of the AuNP extract was 43.1 μg/mL in T24 cells and 53 μg/mL in PC-3 cells. The difference in IC50 values was attributed to the malignant degree of both cell lines, where PC-3 cells are more malignant than T24 cells [194]. In 2018, an in vitro cytotoxicity study on AuNPs biosynthesized by curcumin showed that they had much potential against prostate cancer cells [195].

In 2000, Cytimmune, a United States company, found that AuNPs could attach anticancer drugs and transport them through the bloodstream, delivering them to tumors. In this regard, Cytimmune and AstraZeneca signed an agreement to investigate the feasibility of a new cancer treatment utilizing AuNPs. Following this agreement, CYT-6091 was tested as the first tumor-targeted nanomedicine. CY-6091 consists of tumor necrosis factor-α (TNF) covalently connected to AuNPs. Although CY-6091 has not been certified for systemic use due to its toxicity, Cytimmune agreed, along with another company, to supply newly designed cancer medication based on AuNPs [196].

In 2016, Mukherjee and associates used the extract of *Peltophorum pterocarpum* (PP) leaves to fabricate monodispersed AuNPs for DOX delivery and assessed the efficacy of the nanoformulation in C57BL6/J female mice. Compared to DOX alone, administration of DOX-loaded AuNPs (b-Au-PP-DOX) significantly reduced tumor growth in an in vivo model. They also discovered that nanoconjugated DOX (b-Au-PP-DOX) was more rapidly taken up and released by tumor-bearing cells than standard DOX. After 7 days of intraperitoneal injections, the animals showed no marked changes in their hematology, serum clinical biochemistry, or histopathology. Their findings reveal that the developed nanoplatform could be utilized in the near future as an alternative cost-effective therapeutic modality against cancer [197]. Various green synthesized AuNPs along with their sources and anticancer outcomes against different cancers, are listed in Table 1.

## 6. Challenges and Opportunities

The fate and behavior of AuNPs in the environment and biological systems challenge their application in cancer therapy and depend on functional features, including size, shape, surface charge, dispersity, and SPR [42]. It has been evidenced that AuNPs are degraded in the liver and hepatocytes and excreted in feces and urine. Gold accumulation in the kidney, liver, and blood plasma decreases over time [199]. AuNPs smaller than 20–30 nm are swiftly eliminated from circulation by the excretory system, such as kidneys; hence, they do not accumulate in the body tissue, whereas particles bigger than 200 nm are absorbed by the phagocytic system [200,201,202]. The concentration, aggregation, and circulation period of AuNPs all affect the effectiveness of drug delivery systems. Commonly, AuNPs are nontoxic at low concentrations, while they become toxic when the incubation time is prolonged or the concentration is increased [202]. In the drug delivery system, the accurate finding of the target cell has a vital role in the efficacy of this therapy method. The accurate design of ligands and antibodies causes the selective recognition of target cells by NPs containing anticancer medicines. This matter increases the targeted delivery and bioavailability at the site of action. It causes lower drug concentrations in nontarget organs, thus reducing the formulation’s toxicity to a minimum level, while unprincipled designing of ligands can cause toxicity for other organs. In this regard, this processing, in turn, results in a rise in the finished product’s cost. Another barrier to AuNPs’ delivery in cancer treatment is the presence of physiological obstacles between NPs and carcinoma cells, including the microvessel wall, extracellular matrix, and plasma membrane [203]. Due to the low convective transport driving force, the movement of NPs is complex in the interstitial region of solid tumors. Therefore, sites with insufficient or no permeability microvessels also exist, resulting in heterogeneity in the distribution of NPs [203,204].

Reaction conditions cannot be changed easily enough to allow the use of green reducing agents in AuNPs synthesis. However, while changes in concentration have an effect, the reduction potential is just as essential and can be greatly influenced by the reducing agent itself. Since green and nontoxic reduction agents are too weak to generate high-quality metal NPs, the toxicity of reductants has also been recognized as a problem. The development of stronger green reducing agents or introducing more effective reaction conditions for weaker reducing agents is indeed an open and crucial challenge in producing high-quality AuNPs without the presence of toxic substances [205]. Although diverse literature is available on the promising anticancer effects of metallic NPs, their mechanisms of action are not fully clear. Green synthesized AuNPs have demonstrated a significant cytotoxicity effect against various cell lines in vitro and have shown no considerable toxicity in healthy cell lines, indicating their biocompatibility. Nevertheless, these factors need to be studied extensively in in vivo and ex vivo models to obtain in-depth knowledge of pharmacokinetics and toxicity profiles of biosynthesized AuNPs. In addition, in the green synthesis of NMs mediated by plants, choosing the proper plant species is crucial because some plants can be toxic to healthy cells. The presence of harmful phytocompounds in certain plants, such as the presence of cobalt in iron in Juglans regia, *Rosa damascena*, and calcium in *Anethum graveolens*, *Juglans regia*, and *Caccinia macranthera*, can result in the release of trace elements from metal oxide and metal biosynthesized NPs, which can result in raised oxidative stress that is related to an increased risk of cancer initiation [206]. Using nontoxic reducing agents and little or no solvent to achieve high yields of single-sized AuNPs is also a difficult but worthwhile goal. In order to reach this aim and guarantee a greener future for AuNPs in research and industries, it is evident that wider discussions on achieving sustainable NPs are required, leading to a deeper appreciation among nanoparticle chemists of the advantages of sustainable plant-mediated synthetic approaches [205]. Another serious limitation is the protein corona effect in vivo, which ultimately compromises the dependability of NPs [207]. Overcoming these limitations represents important opportunities to transform how metal NPs are fabricated. More potent green-reducing agents must be developed to fabricate high-quality AuNPs without toxic effects.

It has been established that utilization of solvent generates all of the waste from NP syntheses via arrested participation. To tackle this challenge, developing entirely novel synthesis methods that decrease solvent use and eliminate size sorting and post-processing of the product seems necessary to reduce waste [205]. In addition, investigations should be carried out on the mechanism of eliminating targeted cancer cells and the fate of AuNPs in the body. Right now, despite improvement and the curative properties of AuNPs, deployed NMs cannot be removed entirely from patients’ blood. However, the detox of NPs from patients’ bodies using a “Nanogold detoxification machine” in 2013 [208] was recommended by Shahidi Bonjar and coworkers. According to their observations, the detoxifying device is based on a “hemodialysis machine”, which would aid in the efficacy of therapy by AuNPs for cancers and avoid AuNPs’ accumulation in nontarget tissues or organs following therapy.

Another strategy would be the functionalization of plant-based AuNPs with synthetic polymers to enhance their targeting efficacy. It has been established that the PEGylation of AuNPs resulted in larger particle size, decreased zeta potential value, and exhibited a completely safe profile for biomedical application [209]. Nevertheless, the commercialization of AuNPs on a large scale requires careful consideration of the selected biological components. Simple, cost-effective, environmentally friendly, and easily scaleable methods should be designed, as should parameters for controlling the morphology and size of NMs. The biochemical mechanisms involved in nanoparticle fabrication must be understood to maximize the biological system’s potential. After further justifying the current in vitro and in vivo findings via clinical trials in animal studies, green synthesized AuNPs can be used as an alternately formulated chemotherapeutic drug to treat or detect various cancers.

## 7. Conclusions

The usage of green synthesized AuNPs in the literature mainly focuses on evaluating their anticancer effects. In this context, more studies should be carried out regarding imaging, especially its usability in wearable sensor systems. Yet, considering the studies published most recently in the literature, it is clear that the future of green-synthesized AuNPs is very bright as they are much more biocompatible and cost-effective than chemically synthesized AuNPs. Further in vitro/in vivo/ex vivo experiments are warranted for preclinical and clinical assessments of green synthesized AuNPs for early diagnosis and treatment of cancer. These therapeutic modalities might replace chemically synthesized AuNPs in cancer theranostic applications soon. Broader discussions regarding the sustainability of plant-based AuNPs lead to a deeper appreciation among NP chemists who are willing to work toward this goal and guarantee a greener future for AuNPs in research, industries, the marketplace, and more importantly, cancer therapy.

## Figures and Tables

**Table 1:** Anticancer activity of AuNPs using green material against various cancers.

Type of Extract	Plant Material	Particle Size of AuNPs	Cell line/Exposure Time (h)	Outcome (IC_50_)	Ref.
Aqueous extract	*Cynodon dactylon*	22–34 nm	MCF-7/24	31.34 μg/mL	[148]
	*Dendrobium officinale*	30 nm	HepG2/24	Maximum inhibition at 200 μg/mL	[161]
	*Cystoseira baccata*	8.4 nm	HT-29/48	49.61 µM	[168]
			Caco-2/48	79.03 µM	
	*Siberian ginseng*	200 nm	B16/24	CC50 = 10 μg/mL	[191]
Aqueous leaf extract	*Commiphora wightii*	20.2 nm	MCF-7/24	66.11 μg/mL	[149]
	*Albizia lebbeck*	20–30 nm	HCT-116/24	48 μg/mL	[171]
	*Argemone mexicana*	20–40 nm	HCT-15/24 and 48	20.53 μg/mL	[173]
				12.03 μg/mL	
	*Prosopis farcta*	25 nm	HT-29/72	419.7 µg/mL	[174]
	*Moringa oleifera*	10–20 nm	A549/24	98.46 μg/mL	[181]
			SNO/24	92.01 μg/mL	
	*Phragmites australis*	18 nm	A549/72	129 μg/mL	[182]
	*Centaurea behen*	<50 nm	THP-1/24	25 μg/mL	[183]
	*Cannabis sativa*	18.6 nm	MOLT-3 TALL-104/24 (Jurkat, Clone E6-1)/48 J.RT3-T3.5/72	329 μg/mL	[185]
				381 μg/mL	
				502 μg/mL	
				567 μg/mL	
	*Curcumae*	8–25 nm	PA-1/48	153 μg/mL	[188]
	*Kwangsiensis*		SW-626/48	166 μg/mL	
			SK-OV-3/48	204 μg/mL	
Leaf extract	*Mentha Longifolia*	36.4 nm	MCF-7/48	264 μg/mL	[150]
			Hs 578Bst/48	269 μg/mL	
			Hs 319.T/48	224 μg/mL	
			UACC-3133/48	201 μg/mL	
	*Moringa oliefera*	35–51 nm	MCF-7/24	67.92 μg/mL	[151]
	*Mimosa pudica*	12.5 nm	MCF-7/48	6 μg/mL	[154]
			MDA-MB-231/48	4 μg/mL	
	*Catharanthus roseus*	25–35 nm	HeLa/24	5 μg/mL	[157]
	*Alternanthera Sessilis*	20–40 nm	HeLa/24	Concentration-dependent cell death (10–15 μg/mL)	[155]
	*Zataria multiflora*	10–42 nm	HeLa/48	100 μg/mL	[158]
	*Abutilon indicum*	1–20 nm	HT-29/24 and 48	210 μg/mL	[175]
				180 μg/mL	
	*Sasa borealis*	10–30 nm	AGS/24	120 μg/mL	[192]
Stems	*Atriplex halimus*	2–10 nm	MCF-7/48	47.03 μg/mL	[152]
	*Chenopodium amperosidies*	~40 nm		22 μg/mL	
Pulp extract	Dragon fruit	10–20 nm	MCF-7/24	80% inhibition at highest dose (500 μg/mL after 48 h exposure)	[153]
			MDA-MB-231/48	No significant effect	
	*Annona muricata*	20–30 nm	Hep2, 24	10.94 μg/mL	[165]
Ethanolic, aqueous	*Taxus baccata*	<20 nm	MCF-7/48 and 72	Maximum cell mortality in Hela cells, followed by MCF-7 and Caov-4.	[159]
			Caov-4/48 and 72		
			HeLa/48 and 72		
Isolated from seaweed	*Fuciodan*	31 nm	HepG2/24	Maximum inhibition at 100 μg/mL	[162]
Biomass	*Cordyceps*	15–20 nm	HepG2/24	10 and 12.5 μg/mL	[163]
	*Militaris*				
Essential oil	*Ferula persica*	37.05 nm	CT26/24	0.0024 mg/mL	[169]
Leaf powder	*Cassia*	57 nm	Col320/24	Maximum inhibition at 75 μg/mL	[170]
	*tora*				
Fruit body	*Ganoderma lucidum*	1–100 nm	HT-29/24	84.58 μg/mL	[176]
Aqueous peel extract	*Musa paradisiaca*	50 nm	A549/24	58 μg/mL	[179]
Aqueous flower extract	*Tribulus terrestris*	10–15 nm	THP-1/72	468 μg/mL	[184]
Plant extract	*Scutellaria barbata*	0.4–1 μm	PANC-1/24, 48, and 72	Maximum inhibition at 100 μg/mL	[189]
	*Marsdenia tenacissima*	50 nm	A549/72	15 μg/mL	[178]
Seed extract	*Elaeocarpus ganitrus*	30.34 nm	PC-3	64.23 μg/mL	[141]
	*Trachyspermum ammi*	16.63 nm	HepG2, 48	92.453 µg/mL	[164]
Aqueous rhizome extract	*Curcuma wenyujin*	200 nm	A498/24	CC50 = 25 μg/mL	[190]
			SW-156/24	CC50 = 40 μg/mL	
Ethanolic extract	*Vitex negundo*	30 nm	AGS/24, 48 and 72	15 and 20 μg/mL	[193]
Extract and fractions	*Brazilian red propolis*	8–15 nm	T24/24	AuNPs prepared from extract showed the highest cytotoxicity	[194]
			PC-3/24		
Aqueous root extract	*Crocus sativus*	15 nm	PC-3/24 and 48	AuNPs prepared from curcumin showed effective cytotoxicity at	[195]
-	*Vibrio alginolyticus*	100–150 nm	HCA-7/24	15 μg/mL	[198]
	Curcumin	5–25 nm	MCF-7/36 MDA-MB-231/36	Combinations of AuNPs showed higher anticancer activity as compared to individual AuNPs	[142]
	Turmeric	3–20 nm			
	Quercetin	15–60 nm			
	Crocin	4–10 nm	MCF-7/24	1.8 mg/mL	[147]
			MCF-7/43	1.2 mg/mL	

## Data Availability

Data sharing is not relevant to this paper as no new data were created or analyzed in this study.

## Abbreviation

BRP	Brazilian red propolis
NPs	nanoparticles
PBMCs	peripheral blood mononuclear cells
CNTs	carbon nanotubes
NMs	nanomaterials
MNPs	metal nanoparticles
NIR	near-infrared
SEM	scanning electron microscopy
TEM	transmission electron microscopy
DLS	dynamic light scattering
CT	computed tomography
CDI	color Doppler ultrasound imaging
SPR	surface plasmon resonance
siRNA	short interfering ribonucleic acid
HPV	human papillomavirus
RME	receptor-mediated endocytosis
PSA	prostate-specific antigen
CEA	carcinoembryonic antigen
ctDNA	circulating tumor DNA
ctRNA	circulating tumor DNA
PET	positron emission computed tomography
MRI	magnetic resonance imaging
AuNPs	gold nanoparticles
rGO	reduced graphene oxide
LOD	limit of detection
LDH	lactate dehydrogenase
HCC	hepatocellular carcinoma
TUNEL	terminal deoxynucleotidyl transferase biotin-dUTP nick end labeling
DPPH	2,2-diphenyl-1-picryl-hydrazyl-hydrate
ROS	reactive oxygen species
DAPI	4′,6-diamidino-2-phenylindole
Ao/EtBr	Acridine orange/Ethidium bromide
MTT	3-(4,5-dimethylthiazol-2-yl)-2,5-diphenyl-2H-tetrazolium bromide
IC50	half-maximal inhibitory concentration
PI	propidium iodide
CCK8	Cell Counting Kit 8
TUNEL	terminal deoxynucleotidyl transferase dUTP nick end labeling
GSH	glutathione
SPIONs	superparamagnetic iron oxide nanoparticles
RT-PCR	reverse transcription-polymerase chain reaction
FTIR	Fourier transform infrared spectroscopy
UV-Vis	ultraviolet-visible
GC-MS	gas chromatography-mass spectrometry
PARP	poly (ADP-ribose) polymerase
HRTEM	high-resolution transmission electron microscopy
XRD	X-ray diffraction
EDX	energy-dispersive X-Ray analysis
XPS	X-ray photoelectron spectroscopy
DPPH	2,2-diphenylpicrylhydrazyl
CC50	50% cytotoxic concentration
qPCR	quantitative polymerase chain reaction
FESEM	field-emission scanning electron microscopy
EDS	energy-dispersive spectroscopy
IFN-γ	interferon gamma
IFN-α	interferon alfa
TNF-α	tumor necrosis factor-alpha
WBC	white blood cells
HUVECs	human umbilical vein endothelial cells
